# Melatonin Attenuates Sepsis-Induced Acute Lung Injury Through Improvement of Epithelial Sodium Channel-Mediated Alveolar Fluid Clearance Via Activation of SIRT1/SGK1/Nedd4-2 Signaling Pathway

**DOI:** 10.3389/fphar.2020.590652

**Published:** 2020-12-11

**Authors:** Jing Li, Longfei Liu, Xiaojun Zhou, Xianzhou Lu, Xianrong Liu, Guojuan Li, Jianwu Long

**Affiliations:** ^1^ Department of Endocrinology, Affiliated Nanhua Hospital, University of South China, Hengyang, China; ^2^ Department of Hepatobiliary Surgery, Affiliated Nanhua Hospital, University of South China, Hengyang, China

**Keywords:** melatonin, sepsis, acute lung injury, epithelial sodium channel, silent information regulator 1

## Abstract

Acute lung injury is characterized by alveolar vascular barrier injury, and protein-rich pulmonary oedema. Alveolar fluid clearance is closely related to the prognosis of patients with acute lung injury. Melatonin has been shown to have a protective effect on multiple organ injury induced by sepsis. In this study we investigated the effect of melatonin on alveolar fluid clearance (AFC) and explored its potential mechanisms in sepsis-induced acute lung injury. The cecal ligation and puncture was adopted to establish mouse sepsis model. Morphological changes of lung tissues with the hematoxylin staining were observed. AFC and lung wet/dry weight ratio were measured to assess pulmonary edema. Inflammatory mediators in bronchoalveolar lavage fluid were analyzed via enzyme-linked immunosorbent assay. NAD^+^/NADH and SIRT1 activity were measured by colorimetric assay kit. The protein expressions of epithelial sodium channel (ENaC), silent information regulator1 (SIRT1), SGK1 and Nedd4-2 were immunoblotted by western blot *in vivo* and *in vitro*. The distribution of α-ENaC and SIRT1 was detected by immunofluorescence. We found that melatonin attenuated sepsis induced lung injury, improved survival rate, enhanced alveolar fluid clearance, improved SIRT1 activity, increased protein expressions of SIRT1 and ENaC, and activated SGK1/Nedd4-2 pathway. Furthermore, SIRT1 inhibitor EX527 counteracted the effects of melatonin on alveolar fluid clearance and ENaC. These results revealed that melatonin enhanced ENaC-mediated AFC via the SIRT1/SGK1/Nedd4-2 signaling pathway. Our study demonstrated that melatonin might provide a novel therapeutic strategy for sepsis-induced acute lung injury.

## Introduction

Acute lung injury (ALI)/acute respiratory distress syndrome (ARDS) is characterized by diffuse alveolar injury, increased lung permeability and protein-rich pulmonary oedema (Fan et al., 2018). Several etiological factors associated with ARDS have been identified as sepsis, pneumonia and trauma (Dushianthan et al., 2011). In sepsis-induced ALI, disordered inflammation causes damage to alveolar epithelial and endothelial cells, leading to alveolar effusion (Matthay et al., 2017). Alveolar fluid clearance (AFC) is a main function of alveolar epithelium. In most ARDS patients, the ability to clear excessive alveolar fluid was impaired, and the decrease of AFC was associated with higher mortality (Ware and Matthay, 2001; Zeyed et al., 2012).

The epithelial sodium channel (ENaC) is a heterotrimer protein, which plays an important role in reducing pulmonary edema and promoting AFC (Hummler and Planès, 2010). ENaC consists of three subunits, located in the apical membrane of alveolar epithelial cells. The *α* subunit is an essential subunit for the formation of functional ENaC, while *β* and *γ* subunits promote the activity of channels. The clearance rate of alveolar fluid is related to the active transport of Na^+^ in alveolar epithelium through the apical ENaC and basolateral Na^+^–K^+^-adenosine triphosphates (Na, K-ATPase). Osmotic pressure induced by Na^+^ transport drives water reabsorption (Sartori and Matthay, 2002; Matalon et al., 2015). Therefore, ENaC is considered to be the rate limiting factor of AFC in acute lung injury. The activity of ENaC is regulated by kinases such as serum and glucocorticoid regulated kinase (SGK). All SGK isoforms have been shown to promote ENaC activity, SGK1 was the most potent stimulators of ENaC activity and the actions of SGK1 on ENaC activity are the most well defined to date. SGK1 interacts with and phosphorylates ubiquitin ligase neural precursor cell expressed developmentally downregulated 4-like (Nedd4-2), which catalyzes the addition of ubiquitin molecules to proteins and targets them for degradation by the proteasome or recycling via the lysosome. Phosphorylation of Nedd4-2 decreases its binding to ENaC and increases membrane abundance of ENaC channels (Soundararajan et al., 2012).

Melatonin, also known as n-acetyl-5-methoxy-tryptamine, is mainly synthesized in the pineal gland of mammals (Hardeland, 2018). Recent studies have reported that melatonin protected the organ damage induced by sepsis (Esteban-Zubero et al., 2016; Hu et al., 2017). Silent information regulator1 (SIRT1), a NAD^+^ dependent deacetylase, is involved in the organ protection of melatonin (Rahim et al., 2017). However, it is not clear whether SIRT1 signal is involved in the regulation of melatonin on ALI induced by sepsis. The aim of this study was to investigate the therapeutic effect of melatonin on sepsis induced acute lung injury (ALI) and its potential effects on AFC and ENaC. The role of SIRT1 signaling in mediating the protective mechanism of melatonin has also been evaluated.

## Materials and Methods

### Animals

Male C57BL/6 J mice (8–12 weeks) weighing 22–25 g were obtained from Shanghai Experimental Animal Center (China) and housed under a 12 h day/night cycle. Mice were allowed food and water *ad libitum*. All animal procedures were approved by the Ethics Committee of Animal Experiments of the University of South China.

The cecal ligation and puncture (CLP) model was produced as described previously (Matsuda et al., 2009; Qian et al., 2018). Mice were anesthetized by isoflurane (1–1.5%). The abdominal hair of mice was removed by hair removal cream. After abdominal disinfection, the cecum was exposed through a median abdominal incision (about 1.5 cm). The middle segment of cecum was ligated. The cecum was punctured twice with a 20-gauge needle, then the cecum was repositioned into the abdominal cavity, and the abdominal incision was closed. In the sham operation group, the same surgery was performed, but no ligation or puncture was performed. After operation, all mice were subcutaneously injected with saline (3 ml/100 g body weight) to prevent dehydration.

Mice were divided into six groups with 20 mice in each group: (1) sham group, (2) CLP group, (3) CLP + melatonin (CLP+MEL) group, (4) CLP+ melatonin +EX527 (CLP+ MEL+ EX527) group (EX527 is an inhibitor of SIRT1), (5) melatonin (MEL) alone group and (6) EX527 alone group. In the sham group, melatonin group and EX527 group, mice were subjected to sham operation. In the CLP+melatonin group, CLP+melatonin+EX527 group and melatonin group, mice were given melatonin (30 mg/kg) i.p. daily for 3 days before CLP surgery, on the fourth day mice were given one dose of melatonin 1 h before surgery, and mice in the other groups were received an equal volume of solvent. In the CLP+melatonin+EX527 and EX527 group, 1.2 mM EX527 was prepared in phosphate-buffered saline (PBS) for a single intravenous (5 μL) injection at 1 h before administration of melatonin, and mice in the other groups were given an equal volume of PBS. The dosages and routes of melatonin and EX527 were based on the previous studies (Zhang et al., 2016; Chan et al., 2018). The mice were sacrificed at 24 h after CLP operation.

### Alveolar Fluid Clearance

Alveolar fluid clearance determinations were done by measurement of progressive increase in the concentration of alveolar Evans blue dye (Sakuma et al., 2004; He et al., 2019). The lung and trachea were integrally removed after thoracotomy, and then 1 ml saline containing 5% Evans blue labeled albumin was perfused into the lung through trachea, and followed by 2 ml air to ensure that the saline was distributed to the alveolar spaces. The lungs were placed at 37°C and inflated with 100% oxygen for 1 h. The airway pressure was maintained at 7 cm H_2_O. AFC was calculated as follows:

AFC%=[(Vi−Vf)/Vi] × 100%

Vf=(Vi×Ci)/Cf

V is the initial volume (i) and final volume (f) of alveolar fluid. C is the concentration of Evans blue-labeled albumin solution in initial solution (i) and final alveolar fluid (f).

### Histopathological Analysis

The right upper lobes of the lungs were collected at 24 h after CLP operation and fixed in paraformaldehyde for 48 h. After dehydration with graded alcohol and embedding in paraffin, tissues were cut into 5 μm sections. Then the sections were dewaxed by xylene, hydrated and stained with hematoxylin eosin. Pathological degree of hematoxylin and eosin sections were semi quantitatively graded. According to alveolar congestion, lung edema, neutrophil infiltration, vessel wall and alveolar wall thickness, the pathological degree of HE sections were estimated.

### Evaluation of Pulmonary Edema

Pulmonary edema was assessed by dry/wet weight ratio. The middle lobes of the lungs were excised and blotted with a filter paper to absorb the surface water. The weight of lung tissue was measured and recorded as the wet weight (W). The lung tissue was then dried at 65°C in an oven for 48 h, and its dry weight (D) was measured.

### NAD^+^/NADH Assay

The levels of both NAD and NADH in lung tissue lysates were measured by NAD/NADH Assay Kit (Colorimetric) (Abcam) according to manufacturer’s instructions. The assay was read by absorbance at 450 nm. The level of NAD^+^ was calculated by subtracting NADH from NADt (total NAD^+^ and NADH).

### SIRT1 Activity Assay

Weighed lung tissue and cut it into small pieces. Lung nuclear protein was then extracted using a nuclear extraction kit (Epigentek, Farmingdale, NY, USA). After measuring the protein concentration of nuclear extract, SIRT1 enzymatic activity was measured. A SIRT colorimetric assay kit (Epigentek, Farmingdale, NY, USA) was used according to the manufacturer’s instructions. The activity of the SIRT enzyme was measured by reading the absorbance in a microplate reader at 450 nm.

### Measurement of Inflammatory Cytokines

The mice were anesthetized with pentobarbital sodium (50 mg/kg). Trachea and lungs were then exposed. Saline (0.5 ml) was injected into the left lung through an endotracheal catheter and was withdrawn 5 times. After extraction for 5 times, bronchoalveolar lavage fluid (BALF) was collected and centrifuged at 4°C and 1,000 rpm for 15 min. Then the supernatant was collected. TNF-α, IL-6 and IL-1β were analyzed via enzyme-linked immunosorbent assay (R&D Systems, Minneapolis, MN, USA). All were used in accord with the manufacturer’s instruction.

### Western Blot

Mice lung tissues were collected 24 h after CLP surgery. Lung tissue samples were extracted with RIPA lysis buffer (50 mM Tris (pH 7.4), 150 mM NaCl, 1% Triton X-100, 1% sodium deoxycholate, 0.1% SDS, sodium orthovanadate, sodium fluoride, EDTA, and leupeptin) with PMSF and protein phosphatase inhibitor. Then the denatured protein samples (30 μg) were separated by 10% SDS-PAGE, followed by transfer onto 0.45 μm polyvinylidene fluoride (PVDF) membranes (Millipore, billerica, MA, USA). Membranes were blocked for 1 h at room temperature with 5% BSA blocking buffer (Solarbio, Beijing, China). After washing 3 times with Tris buffered saline with Tween-20 (TBST, 20mM Tris-HCl (pH 7.4), 150mM NaCl, 0.1% Tween-20), the membranes were incubated over night at 4°C with one of the following antibodies: α-ENaC (1:1,000, catalog number: PA1-920A, Thermo Scientific, Waltham, MA, USA), β-ENaC (1:1,000, catalog number: 14134-1-AP, Proteintech, Wuhan, China), γ-ENaC  (1:1,000, catalog number: DF8540, Affinity biosciences, Changzhou, china), SGK1 (1:1,000, catalog number:#12103, Cell Signaling Technology, Danvers, MA, USA), Nedd4-2 (1:1,000, catalog number:#4013, Cell Signaling Technology, Danvers, MA, USA) and p-Nedd4-2 (1:1,000, catalog number:#8043, Cell Signaling Technology, Danvers, MA, USA), SIRT1(1:1,000, catalog number: ab189494, Abcam, Cambridge, MA, USA) and GAPDH (1:3,000, catalog number:K200057M, Solarbio, Beijing, China). The membranes were then washed excess antibody and incubated with HRP-conjugated secondary antibodies (1:3,000) at room temperature for 2 h. After washing 3 times, immunoreactivity was visualized by Chemiluminescent Substrate (Thermo Scientific).

### Real-Time PCR Analysis

Total RNA from the lung tissues was extracted by RNAiso plus solution (TaKaRa, Beijing, China). One μg of total RNA was used for synthesizing the cDNA by reverse transcription kit (Thermo Scientific, Rockford, IL, USA). SYBR Premix Ex Taq II (Takara, Beijing, China) was used for real-time PCR. Primers of α-ENaC were as follows (forward)5′-AGCCTAGAGAAG AGGACCCAG-3′ and (reverse) 5′-TCC​TCC​CGG​ACT​GTT​TGA​CT-3′. Primers of β-ENaC were as follows (forward)5′-AGCAGCTTCCTAAACAGCAGGT-3′ and (reverse) 5′-CTCACAGAT GATGCG TTTGGG-3′. Primers of γ-ENaC were as follows (forward)5′-CCCAGGCACCGA CCATTAAG-3′ and (reverse) 5′-CGT​GAA​CGC​AAT​CCA​CAA​CA-3′. All primers were obtained from Genewiz (Genewiz, Suzhou, China). PCR conditions were 30 s at 95°C, followed by 40 cycles of 5 s at 95°C and 20 s at 60°C. Gene expression was normalized with GAPDH as the housekeeping gene, and the relative expression of genes was calculated using the 2^−△△^Ct method.

### Immunofluorescence

Immunofluorescence analysis was performed on the paraffin-embedded sections of lung tissue samples and A549 cells. After deparaffinization and antigen recovery, the sections of lung tissue samples were blocked with 10% donkey serum at room temperature for 1 h. A549 cells were fixed with 4% paraformaldehyde and blocked with 10% donkey serum. The sections of lung tissue samples and A549 cells were then incubated with primary antibody against SIRT1 (1:200, catalog number: ab189494, Abcam, Cambridge, MA, USA) or α-ENaC (1:200, catalog number: PA1-920A, Thermo Scientific, Waltham, MA, USA) overnight at 4°C. The Alexa-Fluor-coupled secondary antibodies (1:200) were used and applied for 1 h at room temperature. Then the sections and cells were counterstained with DAPI and cover slipped, which were followed by analysis using fluorescence microscopy (Leica).

### *In Vitro* Study

Human lung adenocarcinoma cells A549 were obtained from the Cell Bank of the Chinese Academy of Sciences (Shanghai, China). A549 cells were cultured in DMEM medium (Gibco, Waltham, MA, USA) containing 10% (v/v) fetal bovine serum and 1% (v/v) penicillin-streptomycin solution at 37°C in a 5% CO_2_ atmosphere.

### Statistical Analysis

Data are presented as the mean ± standard deviation (SD). Statistical differences were analyzed by one-way ANOVA followed by the post hoc Tukey test for multiple comparisons. The survival analysis was performed by Kaplan Meier estimate and the comparison between groups were performed by the log-rank test. Statistical analysis was performed with GraphPad Prism 6.0 software (GraphPad, San Diego, CA, USA). A value of *p* < 0.05 was considered significant.

## Results

### Melatonin Increased Expression of SIRT1

**FIGURE 1:**
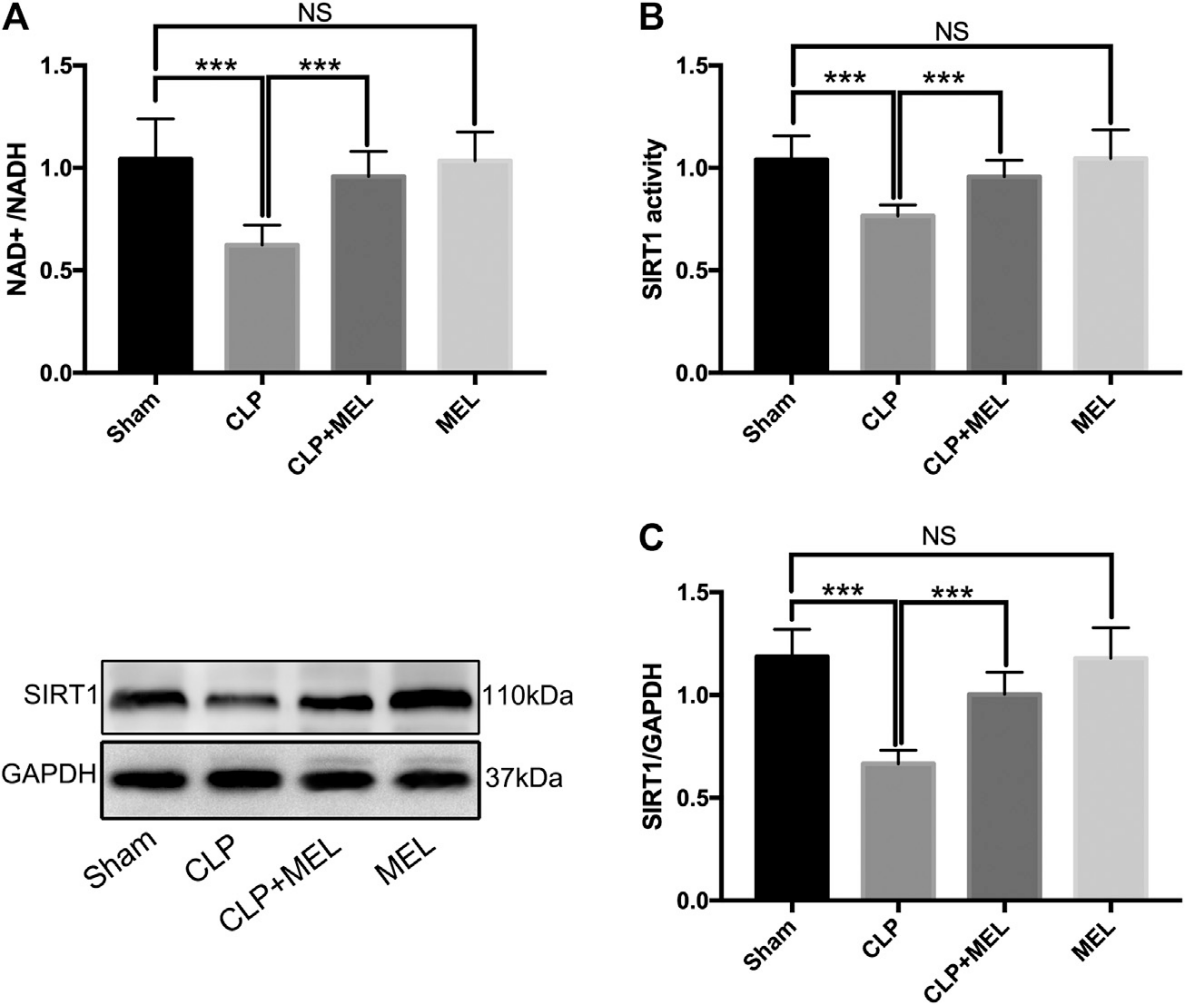
The effect of melatonin on SIRT1. Mice were administrated with melatonin 30 mg/kg daily by intraperitoneal injection for 3 days before CLP or sham operation, on the fourth day mice received one dose of melatonin 1 h before CLP operation. At 24 h after CLP surgery, mice were killed and lung tissues were collected. **(A)** NAD^+^/NADH and **(B)** SIRT1 activity were measured by colorimetric assay kit. **(C)** Protein expression level of SIRT1 was detected by western blot using GAPDH as a loading control. Results obtained by densitometry ratios of target proteins to GAPDH. All values are means ± SD (*n* = 6–8). ***p < 0.001; **p < 0.01; *p < 0.05 (one-way ANOVA followed by Turkey post hoc test).

SIRT1 is a NAD^+^ dependent deacetylase, and involved in the protective effect of melatonin on multiple organs during sepsis. To further confirm the effect of melatonin on SIRT1, we measured NAD^+^/NADH, SIRT1 activity, protein expression level of SIRT1. We found that CLP significantly decreased NAD^+^/NADH and SIRT1 activity compared to sham, but that prior treatment with melatonin prevented this decrease. (Figures 1A,B). Moreover, CLP significantly reduced protein expression of SIRT1 which could be alleviated by pre-treatment of melatonin. (Figure 1C). Administration with Melatonin alone had no significant effect on SIRT1.

### Melatonin Ameliorated CLP-Induced Acute Lung Injury and Improved Survival Rate During Sepsis

**FIGURE 2:**
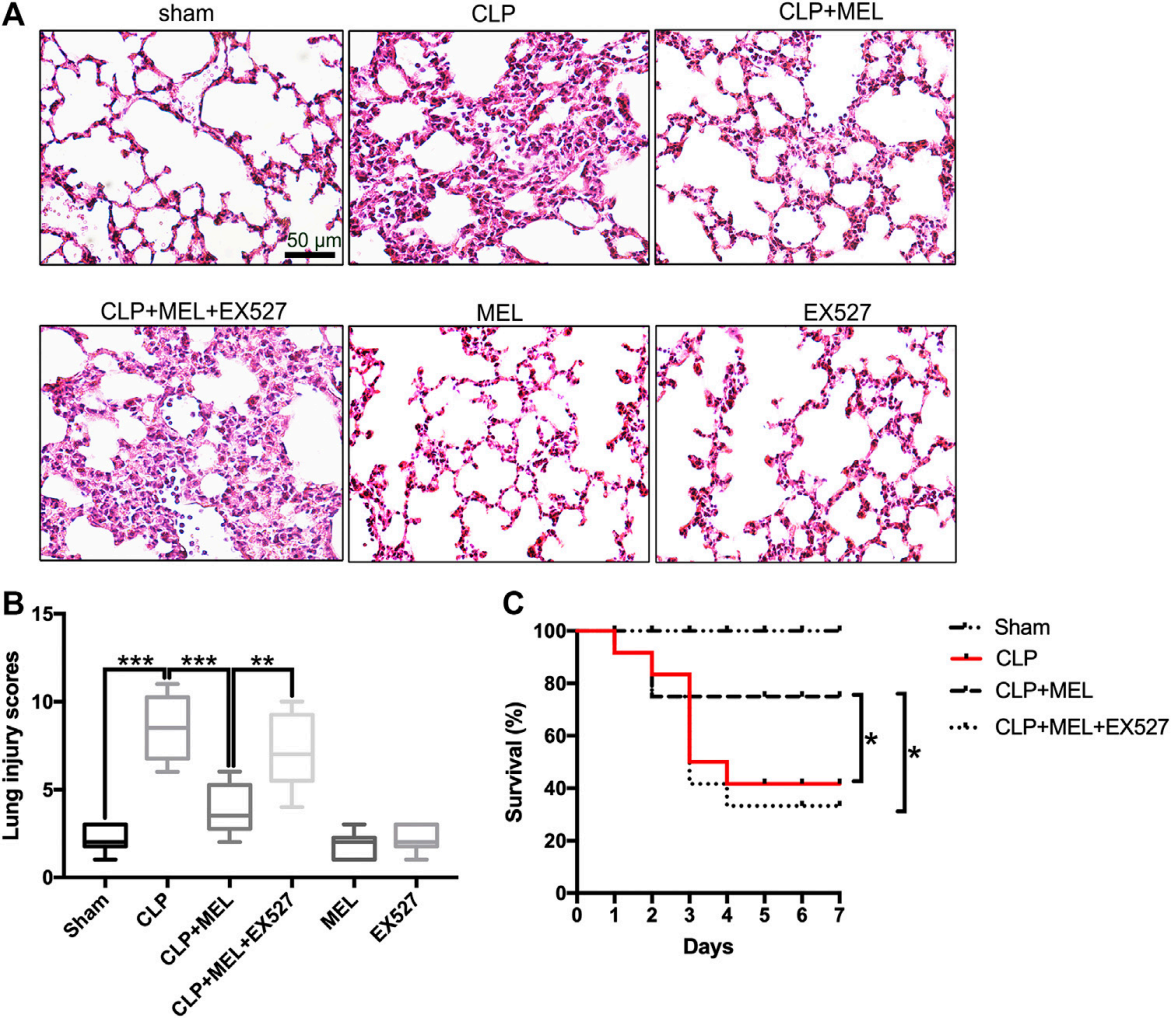
Melatonin protected against lung injury in CLP-induced ALI. Mice were administrated with melatonin 30 mg/kg daily by intraperitoneal injection for 3 days before CLP or sham operation, on the fourth day mice received one dose of melatonin 1 h before CLP operation. EX527 was prepared in phosphate-buffered saline for a single intravenous (5μL) injection 1 h before administration of melatonin. At 24 h after CLP surgery, the effect of melatonin on lung injury was assessed by **(A)** histology. **(B)** Lung damage scores were calculated according to the severity of lung injury. Data were presented as medians and ranges (5th-95th percentile). Kruskal Wallis test was used to evaluate the differences among groups. n = 8, ***p < 0.001; **p < 0.01; *p < 0.05. **(C)** Mice survival rate also was determined, n = 12, ***p < 0.001; **p < 0.01; *p < 0.05 (Pairwise log-rank test).

Previous studies have found that melatonin could protect against organs injury during sepsis (Zhao et al., 2015; Zhang et al., 2019). To further confirm the effect of melatonin on acute lung injury, we observed the morphological changes of lung tissues and evaluated the role of SIRT1 in the lung protection of melatonin. At 24 h post-CLP, compared with sham group, lung tissues in the CLP group were significantly damaged, with hemorrhage, thickening of the alveolar wall and infiltration of inflammatory cells (Figure 2A). Corresponding to this, the lung injury score in CLP group was obvious higher than that in sham Group (Figure 2B). Melatonin significantly attenuated CLP-induced lung damage as shown by the reduction in lung injury score (Figures 2A,B).

In the survival experiment (Figure 2C), CLP resulted in a survival rate of 40% within 7 days, which was much lower than that in the sham group (100%). Mice pre-treatment with melatonin significantly improved survival rate during the observed time. However, the effect of melatonin on lung protection and survival rate was prevented by SIRT1 inhibitor (EX527). These results provided evidence for the protective effect of melatonin in sepsis-induced ALI.

### Melatonin Promoted Alveolar Fluid Clearance and Decreased the Inflammatory Mediators

**FIGURE 3:**
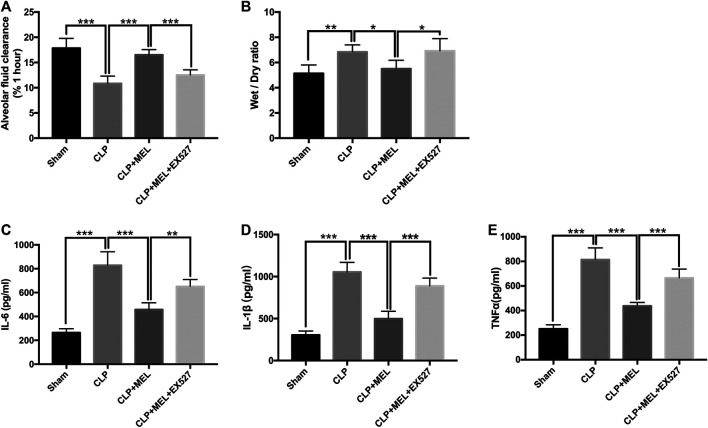
Melatonin increased AFC and alleviated inflammatory mediators in BALF. **(A)** The AFC in ALI mice was determined **(B)** Lung wet/dry weight ratio was tested. The levels of IL-6 **(C)**, TNF-α **(D)** and IL-1β **(E)** in BALF were tested by ELISA kit. All values are means ± SD (*n* = 6). ***p < 0.001; **p < 0.01; *p < 0.05 (one-way ANOVA followed by Turkey post hoc test).

To clarify the effect of melatonin on alveolar fluid balance, we measured lung wet/dry ratios and alveolar fluid clearance. The wet/dry ratio of mice with CLP-induced ALI was elevated while AFC was significantly decreased. Simultaneous treatment with melatonin showed lower wet/dry ratio and enhanced AFC compared with LPS-treated counterparts (Figures 3A,B). The effect of melatonin on alveolar fluid was inhibited by EX527.

A large number of inflammatory mediators are released during sepsis. In this study, we found that the level of TNF-α, IL-6 and IL-1β in the BALF was obviously increased in the CLP group compared with sham group. Melatonin significantly reduced the level of these inflammatory mediators. But the effect of melatonin on inflammatory mediators was inhibited by EX527 (Figures 3C–E).

### Melatonin Increased the Expression of ENaC

**FIGURE 4:**
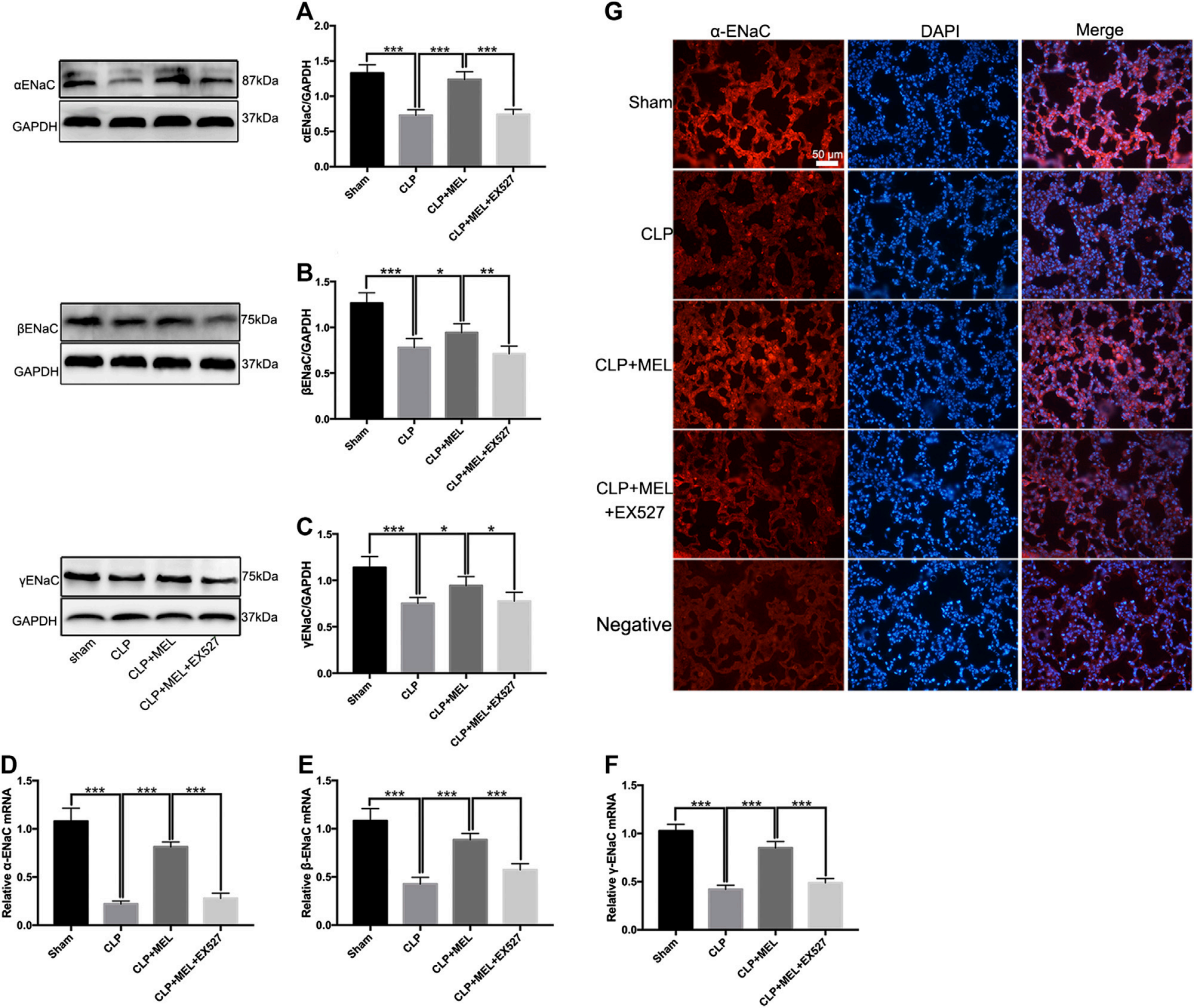
Melatonin enhanced ENaC expression in CLP-induced ALI. The protein expressions of α-ENaC **(A)**, β-ENaC **(B)** and γ-ENaC **(C)** in ALI mouse were determined by western blot using GAPDH as a loading control. Results obtained by densitometry ratios of target proteins to GAPDH. The mRNA expressions of α-ENaC **(D)**, β-ENaC **(E)** and γ-ENaC **(F)** in ALI mouse were tested by real-time PCR. Gene expression was normalized with GAPDH as the housekeeping gene, and the relative expression of genes was calculated using the 2^−△△^Ct method. **(G)** The distribution and protein expression of α-ENaC was examined by immunofluorescence. All values are means ± SD (*n* = 6). ***p < 0.001; **p < 0.01; *p < 0.05 (one-way ANOVA followed by Turkey post hoc test).

ENaC plays a key role in alveolar fluid clearance. To elucidate the mechanism of melatonin on alveolar fluid clearance, we detected the protein and mRNA expression levels of ENaC. Compared with sham group, the protein and mRNA expressions of α, β and γ-ENaC in the lung tissues were significantly decreased in CLP group. Pre-treatment with melatonin markedly attenuated CLP-induced decrease of protein expressions of α, β and γ-ENaC (Figures 4A–C). Melatonin also significantly up-regulated the mRNA expressions of α, β and γ-ENaC in CLP-induced ALI (Figures 4D–F). Among the three subunits, the most significant effect of melatonin was α-ENaC. The protein expression of αENaC was also detected by immunofluorescence (Figure 4G). The results showed that αENaC was localized in cytoplasm and cell membrane. Compared with the sham operation group, the expression of αENaC in CLP group was decreased, while melatonin could alleviate the decrease of αENaC caused by sepsis. However, the increase in ENaC was prevented by EX527. These results further confirmed that melatonin up-regulates ENaC expression in CLP-induced ALI.

### Melatonin Activated the SGK1/Nedd4-2 Pathway in CLP-Induced Acute Lung Injury

**FIGURE 5:**
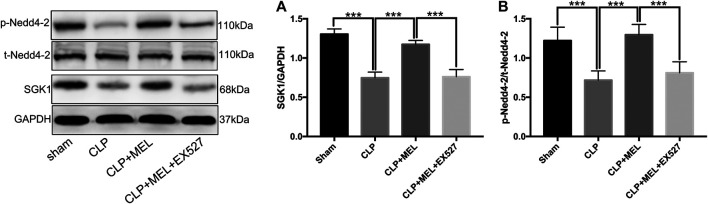
SGK1/Nedd4-2 pathway contributed to the effect of melatonin. The protein expressions of SGK1, p-Nedd4-2 and t- Nedd4-2 were examined by western blot. **(A)** The band intensity of SGK1 was quantitated by normalized for GAPDH **(B)** Phosphorylated Nedd4-2 was quantitated by normalized for total Nedd4-2. The data are presented as the means ± SD (*n* = 6–8). ***p < 0.001; **p < 0.01; *p < 0.05 (one-way ANOVA followed by Turkey post hoc test).

To investigate signaling pathway that involved in the regulation of ENaC. We detected the protein levels of SGK1 and Nedd4-2 in lung tissue homogenates by western blotting. Compared with sham group, the protein expression of SGK1 in the lung tissues was significantly decreased in CLP group. Pre-treatment with melatonin obviously prevented the decrease of protein expression of SGK1 which was reduced by sepsis (Figure 5A). However, the improvement of SGK1 mediated by melatonin was inhibited by EX527 (Figure 5A). Nedd4-2 is a E3 ubiquitin protein ligase, which plays an important role in the regulation of ENaC. Compared with sham group, CLP significantly down-regulated the protein expression of p-Nedd4-2 and the ratio of p-Nedd4-2/t-Nedd4-2 which could be up-regulated by pre-treatment with melatonin during sepsis. The effect of melatonin on the protein expression of Nedd4-2 was also inhibited by EX527 (Figure 5B).

### Melatonin Regulated ENaC Through SIRT1/SGK1/Nedd4-2 Signaling Pathway *in vitro*

**FIGURE 6:**
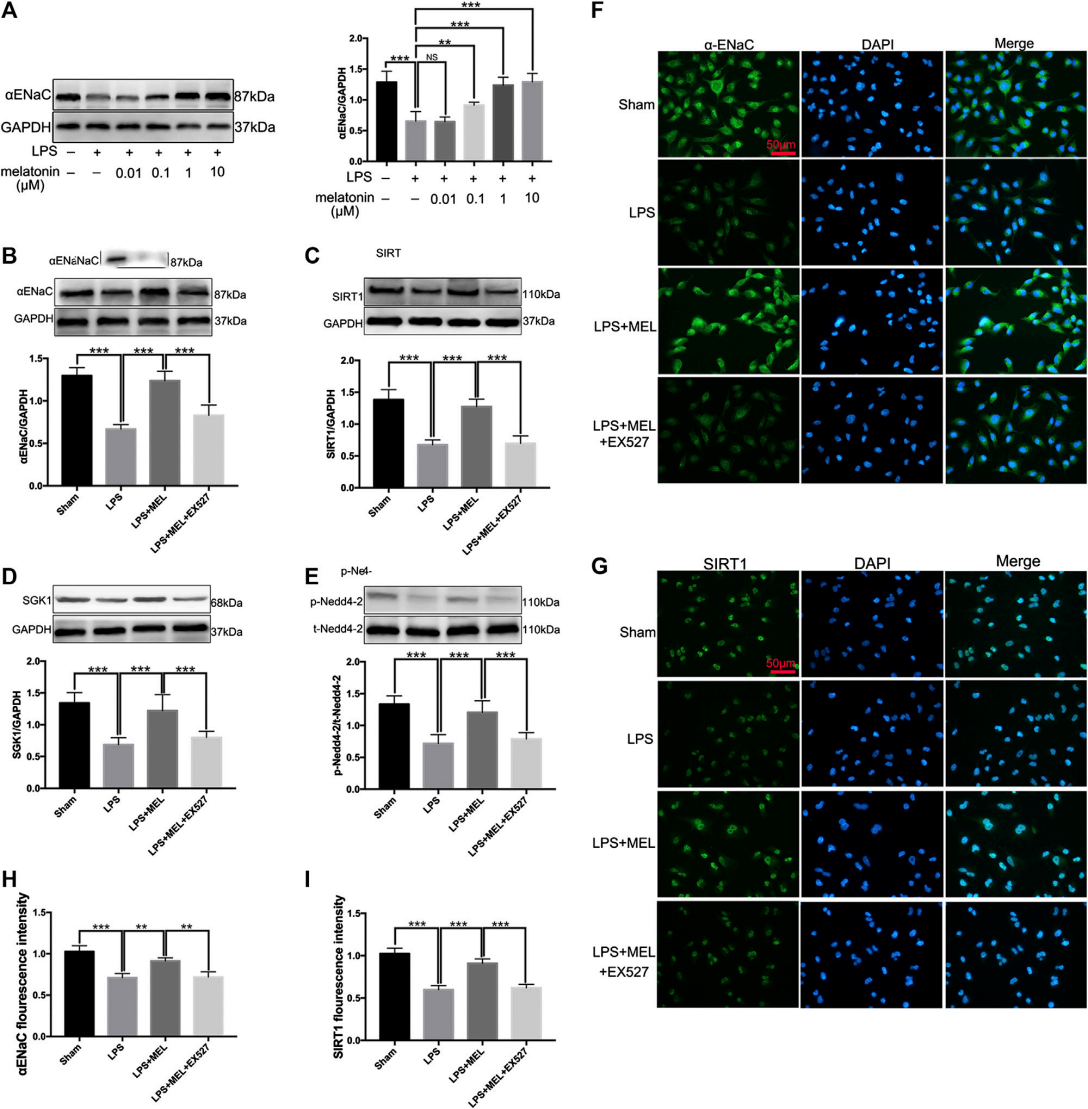
Melatonin up-regulated ENaC protein expression through SIRT1/SGK1/Nedd4-2 pathway in A549 cells. **(A)** A549 Cells were stimulated by LPS (1 μg/ml) with different concentrations of melatonin (10, 100 nM, 1 and 10 μM) for 24 h **(B–G)** A549 cells were treated with LPS in the presence or absence of melatonin (1uM) for 24 h. Inhibitor of SIRT1, EX527 (1uM) was administrated 1 h before melatonin. **(B–E)** The protein expression levels of αENaC, SIRT1, SGK1 and Nedd4-2 were measured by western blot using GAPDH as a loading control. Results obtained by densitometry ratios of target proteins to GAPDH. All values are means ± SD (n = 6). NS, no significance, ***p < 0.001; **p < 0.01; *p < 0.05 (one-way ANOVA followed by Turkey post hoc test). **(F, G)** αENaC and SIRT1 were determined by immunofluorescence **(H, I)** Fluorescence intensity of SIRT1 and α-ENaC was determined by using ImageJ software (version 1.46, NIH, Bethesda, MD, USA, and all values were normalized to sham. All values are means ± SD (*n* = 4). ***p < 0.001; **p < 0.01; *p < 0.05 (one-way ANOVA followed by Turkey post hoc test).

To further verify the effect of melatonin on ENaC, we detected the protein levels of ENaC and related pathway in A549 cells. Firstly, A549 cells were stimulated by LPS (1 μg/ml) with or without different concentrations of melatonin (10, 100 nM, 1 and 10 μM) for 24 h. The results showed that LPS significantly down-regulated the protein expression of αENaC. Melatonin dose-dependently increased protein expression of ENaC (Figure 6A).

Then, A549 cells were treated with LPS in the presence or absence of melatonin (1uM) for 24 h. Inhibitor of SIRT1, EX527 (1uM) was administrated 1 h before melatonin. The protein levels of αENaC, SIRT1, SGK1 and Nedd4-2 were measured by western blot. LPS significantly reduced the protein expressions of αENaC, SIRT1, SGK1 and phosphorylated Nedd4-2, which was reversed by melatonin. However, the effect of melatonin on these proteins could be inhibited by EX527 (Figure 6B–E). The protein expressions of αENaC and SIRT1 in A549 cells were also examined by immunofluorescence. The results revealed that αENaC was mainly located in cytoplasm and cell membrane and SIRT1 was mostly located in nucleus (Figures 6F,G). Melatonin could attenuate the decrease of αENaC protein expression and SIRT1 protein expression mediated by sepsis (Figure 6H,I). These results demonstrated that melatonin up-regulated ENaC protein expression through SIRT1/SGK1/Nedd4-2 pathway.

## Discussion

Infectious etiology is the main cause of ALI/ARDS. CLP model can well simulate animal sepsis model (Piliponsky et al., 2008). Currently, there is no specific treatment for sepsis induced acute lung injury. Here, we established CLP induced acute lung injury to evaluate the effect of melatonin on acute lung injury. We found that pretreatment with melatonin for 3 days before CLP could attenuated sepsis-induced acute lung injury through improvement of ENaC mediated alveolar fluid clearance which maybe through activation of SIRT1/SGK1/Nedd4-2 signaling pathway.

Previous studies have demonstrated that melatonin could protect multiple organs injury during sepsis, and the protective effect of melatonin on organs was mediated by SIRT1, which was an NAD^+^ dependent deacetylase (Bourne and Mills, 2006; Zhao et al., 2015; Zhong et al., 2019). The results in this study revealed that melatonin increased NAD^+^ and SIRT1 expression in CLP-induced acute lung injury. But melatonin alone had no obvious effect on NAD^+^ and SIRT1. This may be due to the lack of NAD^+^ in sepsis, aging and many other pathological conditions. In this study, we used young mice, which were not deficient in NAD^+^ under normal physiological conditions, so administration of melatonin (30 mg/kg) alone in young mice did not improve NAD^+^ level. We also found that melatonin significantly alleviated lung injury and reduced the release of inflammatory mediators in a SIRT1 dependent manner during sepsis.

Acute lung injury is characterized by the destruction of alveolar epithelial barrier, resulting in increased edema formation and impaired alveolar fluid clearance. It is generally believed that the improvement of alveolar fluid clearance is related to the improvement of oxygenation function, the shortening of mechanical ventilation time and the improvement of survival rate (Spinelli et al., 2020). This study revealed that melatonin markedly improved alveolar fluid clearance and reduced pulmonary edema.

Alveolar fluid clearance is dependent on ENaC expressed in alveolar epithelial cells. Improvement of ENaC activity and protein expression, especially on alveolar epithelial surface, can significantly increase alveolar fluid clearance. ENaC is composed of α, β and γ homologous subunits. Among these subunits, the α subunit is the most important one for the transport of sodium ion. In α-ENaC deficient mice, newborn mice were unable to remove respiratory fluid and died within 40 h of birth (Hummler et al., 1996). In this study, melatonin increased the protein expression of α-ENaC and SIRT1 protein in lung tissues and *in vitro*. The effect of melatonin on ENaC was inhibited by SIRT1 inhibitor EX527. These results demonstrated that the improvement of ENaC by melatonin depends on SIRT1 signaling.

SGK1 belongs to a subfamily of serine/threonine kinase, which is called AGC protein kinase (Di Cristofano, 2017). SGK1 is activated in multiple cell signaling pathways and phosphorylation cascades, and plays an important role in ion channels, inflammation and cell survival (Wulff et al., 2002; Borst et al., 2012; Gleason et al., 2015; Manning and Kumar, 2018). The activated SGK1 can phosphorylate the neural precursor cells expressing down regulated protein 4-2 (Nedd4-2), which can negatively regulate ENaC (Debonneville et al., 2001; Snyder et al., 2002). Nedd4-2 ubiquitinates ENaC and promotes ENaC endocytosis. Activation of SGK1 increases p-Nedd4-2, promotes the interaction between Nedd4-2 and 14-3-3 protein, rather than with ENaC, so as to inhibit the ubiquitination and further endocytosis of ENaC, thus improving the cell surface population of ENaC. (Ichimura et al., 2005; Liang et al., 2008). Previous studies showed that reducing Nedd4-2 and ENaC interaction could improve the probability of channel opening (Cook et al., 2002). Zhang found that endotoxin could inhibit the expression and localization of ENaC on alveolar epithelial cells via enhancing Nedd4-2 protein level (Zhang et al., 2017). We also found that melatonin could improve SGK1 expression and inhibit sepsis induced decrease of p-Nedd4-2 protein expression. SIRT1 inhibitor EX527 could eliminate the effect of melatonin on protein expression of SGK1 and Nedd4-2. These results showed that melatonin improved the ENaC through SGK1/Nedd4-2 signaling pathway in ALI induced by sepsis, and the effect of melatonin was mediated by SIRT1.

At present, pharmacological treatment strategies for sepsis are still limited. Although steroid has been used in some patients with sepsis and ARDS, its curative effect is not definite and the use of steroid has obvious adverse effects, such as gastrointestinal bleeding, heart failure, neuromyopathy and complications related to the withdrawal of corticosteroids (Peter et al., 2008; Annane et al., 2018; Duran et al., 2018; Venkatesh et al., 2018; Yao et al., 2020). While the clinical data of melatonin is limited, several animal models of sepsis and our study have shown that melatonin can alleviate sepsis-induced acute lung injury and improve survival during sepsis. The results of this study also suggest that melatonin attenuates sepsis-induced AFC reduction through up regulating ENaC expression via activation of SIRT1/SGK1/Nedd4-2 signaling pathway. In addition, melatonin is safe being devoid of clinically significant side effects (Barlow et al., 2020). These studies suggest that melatonin treatment may be a potential therapy for sepsis-induced acute lung injury.

## Data Availability Statement

The raw data supporting the conclusions of this article will be made available by the authors, without undue reservation, to any qualified researcher.

## Author Contributions

JWL and JL conceived and designed the study. JWL, LL, XZ, GL and XZL performed the experiments. XRL analyzed and validated the data. JWL, JL and LL reviewed and edited the manuscript. All authors read and approved the final manuscript.

## Funding

This research was supported by Guidance Project of Hunan Provincial Health Commission (20201954).

## Conflict of Interest

The authors declare that the research was conducted in the absence of any commercial or financial relationships that could be construed as a potential conflict of interest.
